# Synthesis and Characterization of Nanomaterial Based on Halloysite and Hectorite Clay Minerals Covalently Bridged

**DOI:** 10.3390/nano11020506

**Published:** 2021-02-17

**Authors:** Marina Massaro, Cesar Viseras Iborra, Giuseppe Cavallaro, Carmelo Giuseppe Colletti, Fátima García-Villén, Giuseppe Lazzara, Serena Riela

**Affiliations:** 1 Department of Biological, Chemical and Pharmaceutical Sciences and Technologies, University of Palermo Viale delle Scienze, Ed. 17, 90128 Palermo, Italy; marina.massaro@unipa.it (M.M.); carmelogiuseppe.colletti@unipa.it (C.G.C.); 2 Department of Pharmacy and Pharmaceutical Technology, Faculty of Pharmacy, University of Granada, Campus of Cartuja, 18071 s/n Granada, Spain; fgarvillen@ugr.es; 3 Andalusian Institute of Earth Sciences, CSIC-UGR. Avenida de las Palmeras 4, 18100 Granada, Spain; 4 Department of Physics and Chemistry, University of Palermo, 90128 Palermo, Italy; giuseppe.cavallaro@unipa.it (G.C.); giuseppe.lazzara@unipa.it (G.L.); 5 National Interuniversity Consortium of Materials Science and Technology (INSTM), Research Unit of Palermo, Via G. Giusti, 9, 50121 Florence, Italy

**Keywords:** halloysite nanotubes, hectorite, covalent linkage, synthetic strategy

## Abstract

Halloysite is an aluminosilicate clay with a predominantly hollow tubular structure (HNTs) able to act as a nanocontainer for the encapsulation of several chemicals. However, HNTs possess low affinity for metal ions in their pristine form and they need to be modified for improving their adsorption capabilities. Therefore, to overcome this issue herein we report a straightforward approach for the covalent modification of the external surface of halloysite nanotubes with hectorite clay. Compared to halloysite, hectorite possesses a lamellar structure with higher cation exchange capacity. The covalent linkage between the two clays was verified by several techniques (FTIR spectroscopy, ^13^C CP-MAS NMR, TGA, ζ−potential, DLS, and XRD measurements) and the morphology was imaged by TEM investigations. As proof of concept the adsorption ability of the obtained nanomaterial in comparison to pristine clays was proved using ciprofloxacin and silver ions chosen as models for their different chemical characteristics.

## 1. Introduction

Clay minerals are natural phyllosilicates which present peculiar morphologies that are a challenge for chemical manipulation. Furthermore, their combination at the micro- or nanometric scale plays an important role as scaffold for the development of appealing artificial nanocomposites for several applications ranging from industry to biomedical engineering. Halloysite is an aluminosilicate with a predominantly hollow tubular structure (HNTs) [1]. It is dioctahedral 1:1 clay mineral present in the soil, belonging to the kaolin group. Halloysite possesses different charged surfaces: positive in the inner lumen, where mostly of aluminum hydroxide groups are present; negative on the external surface, which consists in silicon dioxide. HNTs can be selectively functionalized at the inner and/or outer surfaces leading to the synthesis of several interesting nanomaterials [2,3,4,5,6]. The most important feature of HNTs is their empty lumen which acts as a nanocontainer for the encapsulation of several biologically active species [4,7,8]. However, HNTs possess low affinity for metal ions in their pristine form and they need to be modified for improving their adsorption capabilities [9,10]. In the years, several approaches have been exploited but few of them envisage the possibility to delivery simultaneously two or more active ingredients. 

Hectorite (Ht), a natural clay mineral belonging to the smectite group with a general formula of Na_0.3_(Mg,Li)_3_Si_4_O_10_(OH)_2_ possesses a lamellar structure and due to this it shows higher cation exchange capacity [11]. 

Hence, the combination of HNTs and Ht could improve halloysite properties by the development of functional nanoarchitectures which could benefit, in terms of adsorption abilities, thermal stability, solvent resistance and so on, of both HNTs and Ht properties. 

Glotov and Lvov, for example, reported a self-assembly strategy via centhyltrimethyl ammonium bromide onto negative HNTs surface, to obtain MCM-41/HNTs material with enhanced thermal and mechanical stability [12]. An example of chemical mixture between two clay was reported by Mu and Wang [13]. In this case palygorskite and halloysite were combined to improve betain adsortpion. They highlighted that the introduction of halloysite in a palygorskite mixture enhances the physico-chemical properties of palygorskite, such as color performance, heating stability, and solvent resistance. To the best of our knowledge a limited number of publications deals with the preparation of functional materials through the cross-assembly of different components but no one reports the covalent linkage between two clays [14]. This limitation is probably due to the repulsive interactions existing between the negative charges present on the clays external surface. Conversely to the physical combination of clays, a covalent linker among them, could confer to the final nanomaterial new properties, as robustness, which could improve, for example the manufacturing process of pharmaceutical products, by reducing operating cost and improving final product quality.

**Scheme 1:**
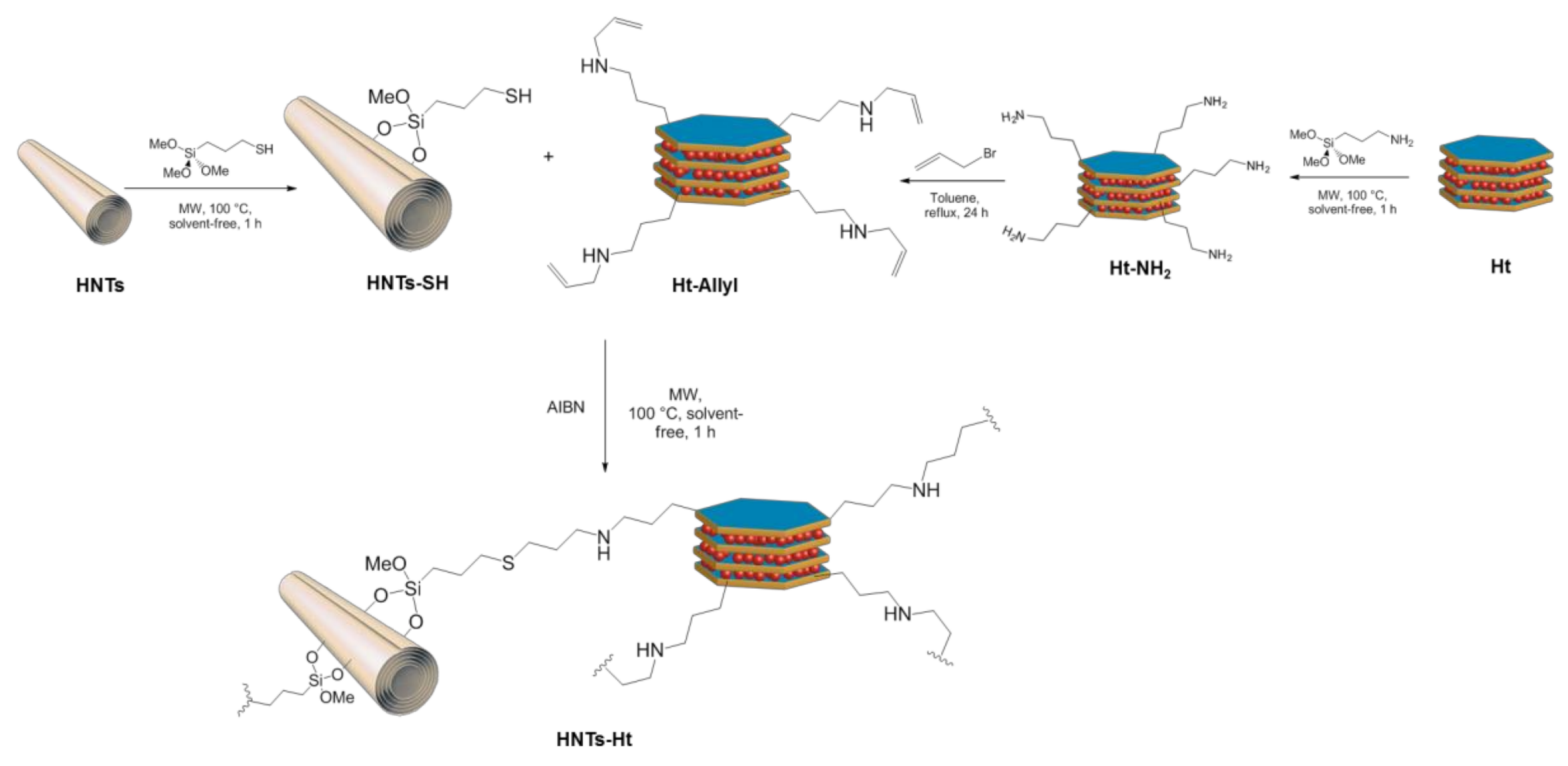
Schematic representation of HNTs-Ht nanomaterial synthesis.

Herein, we report a bottom-up approach to synthetize a multifunctional nanomaterial based on halloysite covalently bridged to hectorite (HNTs-Ht) (Scheme 1), that could highlight the peculiarity of the single clays simultaneously. Indeed, by considering the above-mentioned properties of the two clay minerals, the incorporation of Ht in HNTs external surface is made with a specific goal: to bring or complement missing properties of HNTs, as adsorption ability towards metal ions. The covalent linkage between the two clays was verified by several techniques (FTIR spectroscopy, ^13^C CP-MAS NMR, TGA, ζ−potential, DLS and XRD measurements) and the morphology was imaged by TEM investigations. As proof of concept the adsorption ability of HNTs-Ht compared to pristine clays was proved using two antibacterial agents such as ciprofloxacin and silver ions as models.

## 2. Materials and Methods

All reagents needed were purchased from Sigma-Aldrich and used without further purification.

Hectorite was kindly gifted by Tolsa Group Inc. (Madrid, Spain).

Thermogravimetric analyses were performed on a Q5000 IR apparatus (TA Instruments, New Castle, DE, USA) under a nitrogen flow of 25 cm^3^ min^−1^ for the sample and 10 cm^3^ min^−1^ for the balance. The weight of each sample was ca. 10 mg. Measurements were carried out by heating the sample from room temperature up to 900 °C at a rate of 10 °C min^−1^. 

FTIR spectra (KBr) were recorded with an Agilent Technologies Cary 630 FTIR spectrometer (Agilent Technologies, Santa Clara, CA, USA). Specimens for these measurements were prepared by mixing 5 mg of the sample powder with 100 mg of KBr. FTIR spectrum of the HNTs-Ht nanomaterial was recorded on a JASCO6200 spectrophotometer (software Spectra Manager II). All samples were studied in the 400–4000 cm^−1^ range, with a resolution of 2 cm^−1^.

Microwave-assisted syntheses were carried out with a CEM DISCOVER monomode system (CEM Corporation, Matthews, NC, USA) in a closed vessel.

^13^C CP-MAS NMR spectra were acquired on a Bruker Advance II 400 spectrometer (Bruker, Billerica, MA, USA) operating at 100.63 MHz for ^13^C nuclides and 400.15 MHz for 1 H nuclides equipped with a 4 mm (H–X) double channel CPMAS probe. The samples were placed in a 4 mm zirconia rotor closed with Kel-F caps. The spectra were measured at a MAS speed of 8 kHz, with 2 ms contact time, 3 s delay time and an excitation pulse of 4.7 ms on the 1 H nucleus, and 1024 scans were performed. The Hartmann–Hahn-condition was optimized using an adamantane standard sample, which was also used as an external chemical shift reference.

Transmission electron microscopy (TEM) was performed by means of a FEI Titan G2 60–300 ultra-high resolution transmission electron microscope (FEI, Lausanne, Switzerland) coupled with analytical electron microscopy (AEM) performed with a SUPER-X silicon-drift windowless energy-dispersive X-ray spectroscopy (XEDS) detector. AEM spectra were saved in mode STEM (scanning transmission electron microscopy) with a HAADF (high angle annular dark field) detector. X-ray chemical element maps were also collected.

X-ray powder diffraction (XRPD) analysis was carried out using a diffractometer (X’Pert Pro model, Malven Panalytical, Madrid, Spain) equipped with a solid-state detector (X’Celerator) and a spinning sample holder. The diffractogram patterns were recorded using random oriented mounts with CuKα radiation, operating at 45 kV and 40 mA, in the range 4–60 °2θ. HNTs-SH material was synthetized as reported elsewhere [15].

### 2.1. Synthesis of Ht-NH_2_

A total of 0.500 g of Ht were weighed in an MW test tube provided with a cap, and 1 mL of 3-aminopropyltrimethoxysilane was added dropwise. The mixture was dispersed ultrasonically for 30 min at room temperature and inserted in an MW apparatus at 100 °C under constant stirring for 1 h. The powder was filtered, rinsed with MeOH and dried at 80 °C under vacuum.

### 2.2. Synthesis of Ht-Allyl

In a round bottom flask 0.100 g of Ht-NH_2_ (which correspond to 0.10 mmol of –NH_2_ groups) were dispersed in 10 mL of dry toluene and 100 μL (0.20 mmol, 2 eq) of allyl bromide were added. The mixture was sonicated for 5 min, and then stirred at 110 °C for 24 h. Afterwards, the crude solid was filtered, washed with several aliquots of MeOH and dried overnight at 80 °C under vacuum.

### 2.3. Synthesis of HNTs-Ht

HNTs-SH (50 mg) and Ht-Allyl (50 mg) were weighed in a MW test tube provided with a cap and a catalytic amount of AIBN was added. The mixture was inserted in the MW apparatus at 100 °C, under constant stirring, for 1 h. Successively, the solid was filtered off, rinsed several times with CH_2_Cl_2_ and MeOH and dried at 80 °C under vacuum. 

### 2.4. Loading of Ciprofloxacin on HNTs, Ht and HNTs-Ht

To a dispersion of HNTs, Ht or HNTs-Ht (see infra) in methanol (5 mL), a ciprofloxacin solution (1 mL, 1 × 10^−2^ M in HCl 0.1 N) was added. The suspension was sonicated for 5 min, at an ultrasound power of 200 W and at 25 °C and was left under stirring for 18 h at room temperature. After this time, the solvent was filtered off, under vacuum, and the powder was washed several times with methanol and then dried.

### 2.5. Loading of Silver Ions on HNTs, Ht and HNTs-Ht

To a AgNO_3_ aqueous solution (5 mL, 1 × 10^−2^ M), HNTs, Ht or HNTs-Ht (see infra) were added and the obtained dispersions were left to stir at room temperature for ca. 18 h. After this time, the solvent was filtered off and the powder was washed with water (3 × 5 mL). The powders were dried at 60 °C overnight and the supernatant solutions were analyzed by AgCl test to determine the Ag^+^ ions amount loaded on the clays. In detail, the supernatant solutions were titrated with a standard solution of NaCl (0.01 M) in the presence of dichlorofluorescein, used as indicator. After the endpoint was reached the concentration of Ag^+^ was determined by the following equation:

C_1_V_1_ = C_2_V_2_ (1)

where C_1_ and V_1_ are the concentration of the NaCl solution and the added volume, respectively, while C_2_ and V_2_ are the concentration and volume of Ag^+^ ions in the supernatant solution.

## 3. Results and Discussion

The HNTs-Ht nanomaterial was obtained by reaction between complementary groups grafted to HNTs external surface and modified Ht edges, thiol and allyl, respectively. By means of this synthetic pathway we obtain the best conditions to minimize repulsive interactions that occur between halloysite and hectorite external surfaces (both negatively charged).

### 3.1. Synthesis and Characterization of Ht-Allyl Nanomaterial

Functionalized HNTs by thiol groups was obtained by a procedure reported in literature [15], while Ht was functionalized with allyl units in a two steps procedure (Scheme 1). First, the silanol groups present at the edges of the Ht clay were reacted with an excess of 3-amino propyltrimethoxysilane to obtain an amino functionalized Ht (Ht-NH_2_). This reaction was carried out following experimental procedure previously adopted by us for similar systems [16]. In detail, the grafting of the organosilane at the clay edges was performed by microwave (MW) irradiation at 100 °C, in solvent free conditions, for an irradiation time of 1 h. After work-up, the degree of functionalization of the clay edges, estimated by thermogravimetric analysis (TGA), was 1.03 mmol g^−1^.

The Ht-NH_2_ material was further modified by means of a nucleophilic substitution among the amino groups at Ht edges and allyl bromide, in toluene, at 110 °C for a reaction time of 24 h, to give the compound Ht-Allyl with a degree of functionalization of 2.08 mmol g^−1^ (Scheme 1). The successful functionalization was confirmed by FTIR spectroscopy and TGA.

**Figure 1:**
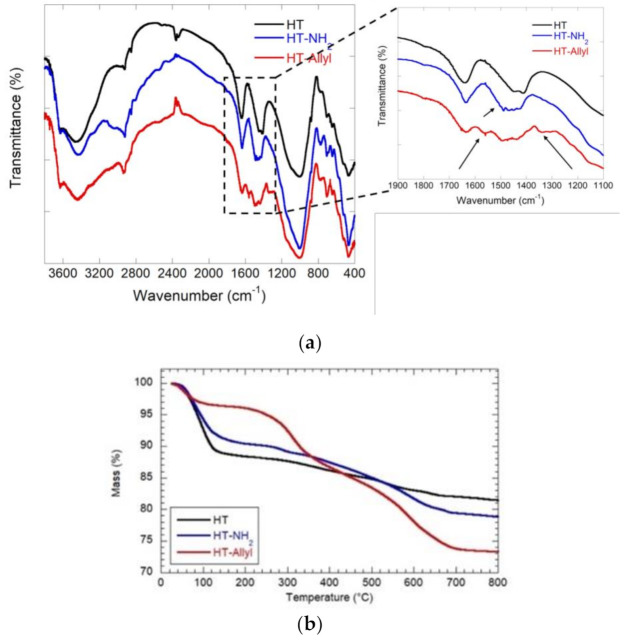
(**a**) FTIR spectra of pristine Ht, Ht-NH_2_ and Ht-Allyl; and TG curves (**b**) for pristine Ht, Ht-NH_2_ and Ht-Allyl.

In Figure 1a are reported the FTIR spectra of Ht-NH_2_ and Ht-Allyl compounds and for comparison that for pristine Ht. The FTIR spectrum of pristine Ht showed all characteristic bands of the clay at ca. 3750−3150 cm^−1^, assigned to the stretching vibration of structural O-H (Mg-O-H and Si-O-H) and adsorbed water. The band at 1635 cm^−1^ is attributed to bending modes of –OH in adsorbed water molecule directly coordinated to the exchangeable cations of the clay mineral and the one at 1020 cm^−1^ is associated to the Si-O-Si stretching vibrations [17]. Compared to pristine Ht, Ht-NH_2_ exhibits, in the FTIR spectrum, the vibration band for C-H stretching of methylene groups around 2980 cm^−1^ and a band around 3200 cm^−1^ due to the vibration stretching bands of the primary –NH_2_ groups of the grafted aminosilane and the vibration band at ~1490 cm^−1^ due to the bending vibrations of the –NH– groups. After reaction with allyl bromide new signals appeared in the FTIR spectrum of the final nanomaterial. In particular is clearly evident the band at ~1560 cm^−1^ that corresponds to the vibration stretching band of C=C bonds and the signal at ~1350 cm^−1^ due to the vinylidene C-H in-plane bending. 

In Figure 1b are reported the thermogravimetric curves for Ht, Ht-NH_2_ and Ht-Allyl materials. The analysis of TG curves allowed us to calculate the mass loss from room temperature to 150 °C (ML_150_) and the residual mass at 700 °C (MR_800_). Based on these results, we calculated the degraded mass at 700 °C (MD_800_) as 100 − (ML_150_ + MR_800_). The thermogravimetric parameters are summarized in Table 1.

According to literature [18], ML_150_ reflects the moisture content of the material. Consequently, this parameter is related to its hydrophilicity. As highlighted in Table 1, the covalent modification of Ht surfaces reduces the hydrophilicity of the nanoclay. In addition, the increase of MD_800_ value in the Ht-NH_2_ and Ht-Allyl materials can be attributed to the presence of organic components proving the successful functionalization of hectorite. 

### 3.2. Synthesis and Characterization of HNTs-Ht Nanomaterial

The covalently linked HNTs-Ht nanomaterial was prepared by reacting the Ht-Allyl nanomaterial with HNTs-SH by AIBN-catalyzed thiol-ene reaction [19] in a 1:1 weight ratio (Scheme 1). The reaction was carried out by MW irradiation, in solvent free conditions, at 100 °C for an irradiation time of 1 h. On the basis of the stoichiometric ratio, allyl groups on Ht were in excess relative to the moles of –SH groups onto HNTs (degree of functionalization of 0.35 and 1.03 mmol g^−1^ for HNTs-SH and Ht-Allyl, respectively), therefore the double bonds that do not react in the thiol–ene reaction can undergo a self-addition reaction, leading to the formation of a coating onto HNTs surface, constituted by a Ht cross-linked network [20].

**Figure 2:**
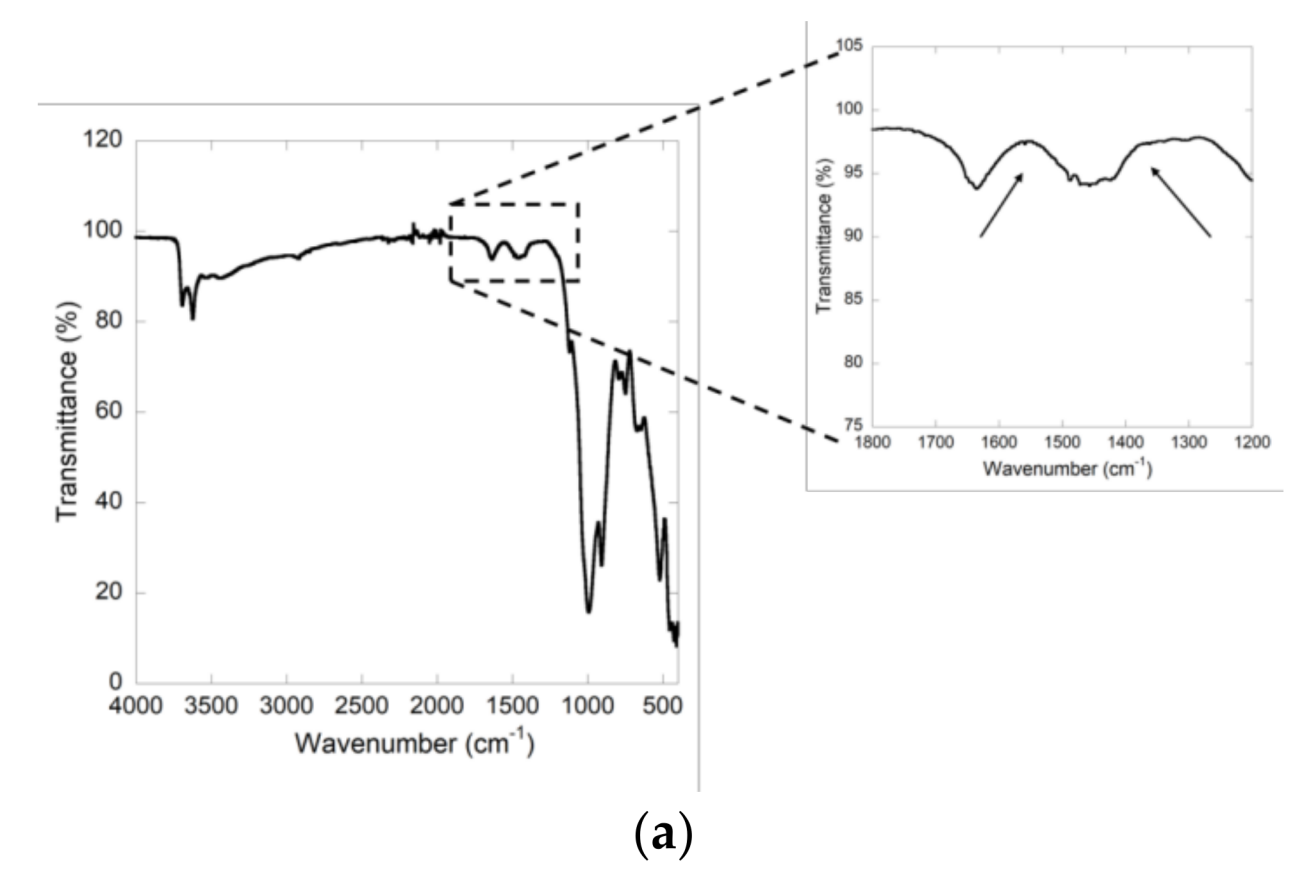

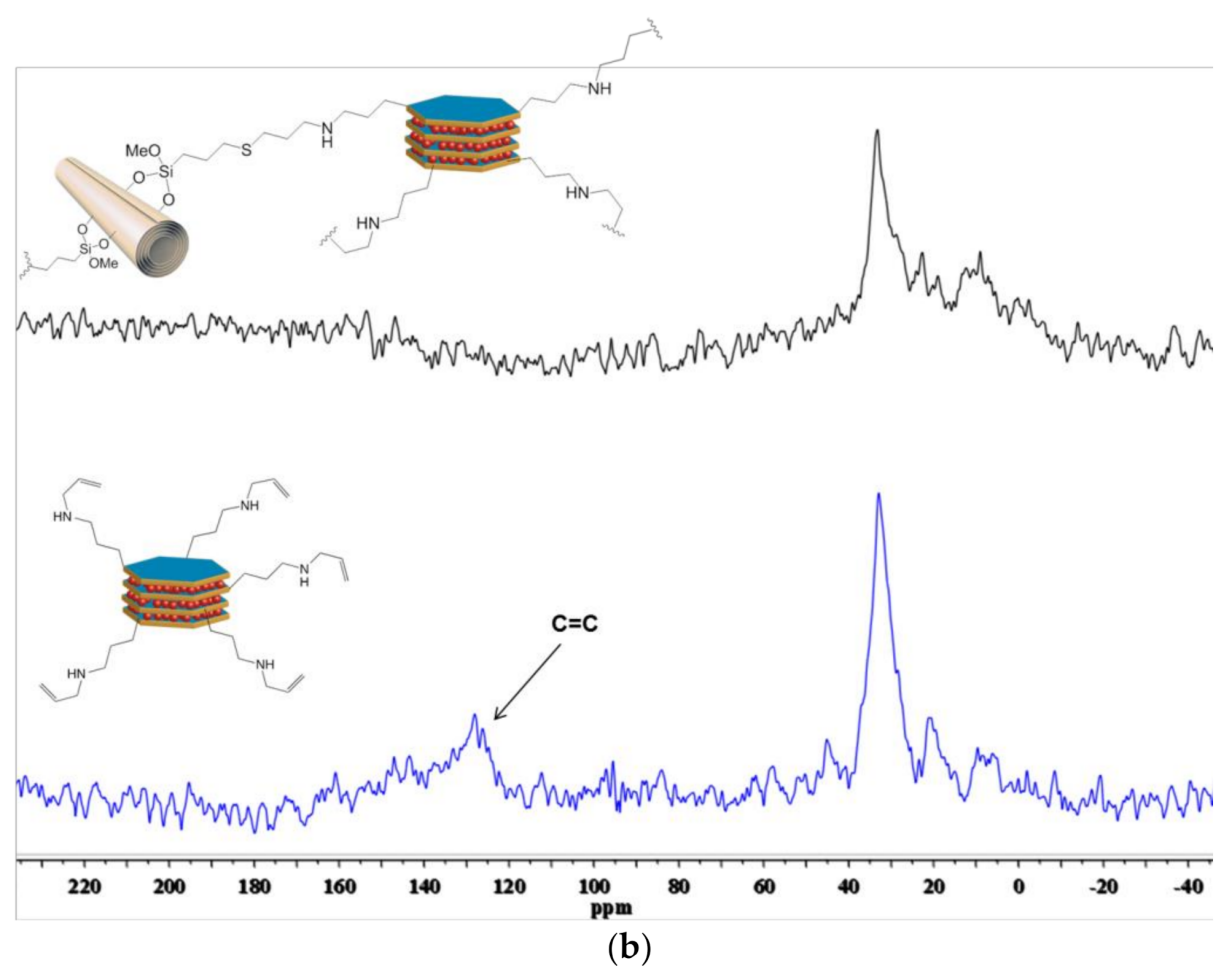
(**a**) FTIR spectrum of HNTs-Ht nanomaterial; (**b**) solid state ^13^C NMR spectra of Ht-Allyl (blue line) and Hal-Ht (black line) nanomaterials.

The HNTs-Ht nanomaterial was characterized by FTIR and ^13^C CP-MAS NMR spectroscopies and TGA. In the FTIR spectrum of HNTs-Ht (Figure 2a) are shown all typical vibration bands of the clays precursors and in addition it showed the disappearance of the bands at ca. 1560 cm^−1^ and 1350 cm^−1^ indicating that the thiol-ene among thiol and allyl groups and the self-addition among different allyl groups reactions occurred.

In Figure 2b, the ^13^C cross-polarization magic angle spinning (^13^C CP-MAS) NMR spectra of the Ht-Allyl and HNTs-Ht nanomaterials are reported. In the solid-state NMR spectrum of Ht-Allyl nanomaterial (blue spectrum), the characteristic signals of the carbons of the allyl groups (−CH=CH_2_) between 150–110 ppm are present, as well as those relative to the carbons of the methylene groups of the organic functionalities in the range 50–20 ppm. In addition, it is also present a signal at ca. 10 ppm attributable to the –CH_2_−Si groups. After the covalent linkage, the solid-state NMR spectrum of HNTs-Ht (black spectrum) shows the disappearance of the signals relative to the allyl groups further confirming the successful covalent linkage between the two clays.

The thermogravimetric curve of HNTs-Ht (Figure S1) evidenced three clear degradation steps in the temperature ranges 25–150, 200–400 and 450–600 °C. As previously reported, the mass loss at 25–150 °C (2.64 wt%) can be attributed to the moisture content. On the other hand, the mass decrease in the range between 200 and 400 °C (6.83 wt%) is related to the organic part of the Hal-Ht hybrid nanomaterial, while the signal change at 450–600 °C (7.95 wt%) is mostly due to expulsion of the interlayer water molecules of halloysite clay nanotubes [3]. 

DLS and ζ−potential experiments were performed to investigate the effects of the synthesis procedure on the aqueous dynamic behavior and the surface charge of halloysite and hectorite clays. 

We observed that pristine hectorite and halloysite possess similar hydrodynamic diameter values (Table 2). The surface modification with allyl and SH groups (for Ht and HNTs, respectively) slightly affects the aqueous dynamic behavior of both nanoclays. In addition, the hydrodynamic diameter of HNTs-Ht is comparable with those of the linked clays. For a correct evaluation of the hydrodynamic diameter, one should keep in mind that these quantities are related to the translational diffusion of the equivalent sphere and therefore are dominated by the larger characteristic size in anisotropic particles. In other words, clustering side-by-side some nanotubes would not affect much the observed values [21]. The constant value of the hydrodynamic size for the linked systems indicates that the network is not extended toward all mass of both materials, instead, small clusters containing a limited number of HNTs/Ht particles are obtained. 

As concerns the ζ−potential, pure Ht and HNTs exhibited similar results. The covalent functionalization generated a decrease of the negative charge of both nanoparticles, while the covalently linked HNTs-Ht nanomaterial evidenced the presence of two populations with different surface charge. The surface charge reduction upon functionalization agrees with the less effective dissociation of the modified surfaces compared to the pristine ones. The presence of two separate values for the hybrid material could indicate the presence of clusters with different Ht/HNTs ratios and, therefore, a surface charge dominated by one of the two clays.

The morphology of the HNTs-Ht nanomaterial was imaged by TEM measurements. According to TEM images, the HNTs-Ht nanomaterial shows the typical nanorod-shaped structure of HNTs and the typical morphology of the hectorite (Figure S2). 

**Figure 3:**
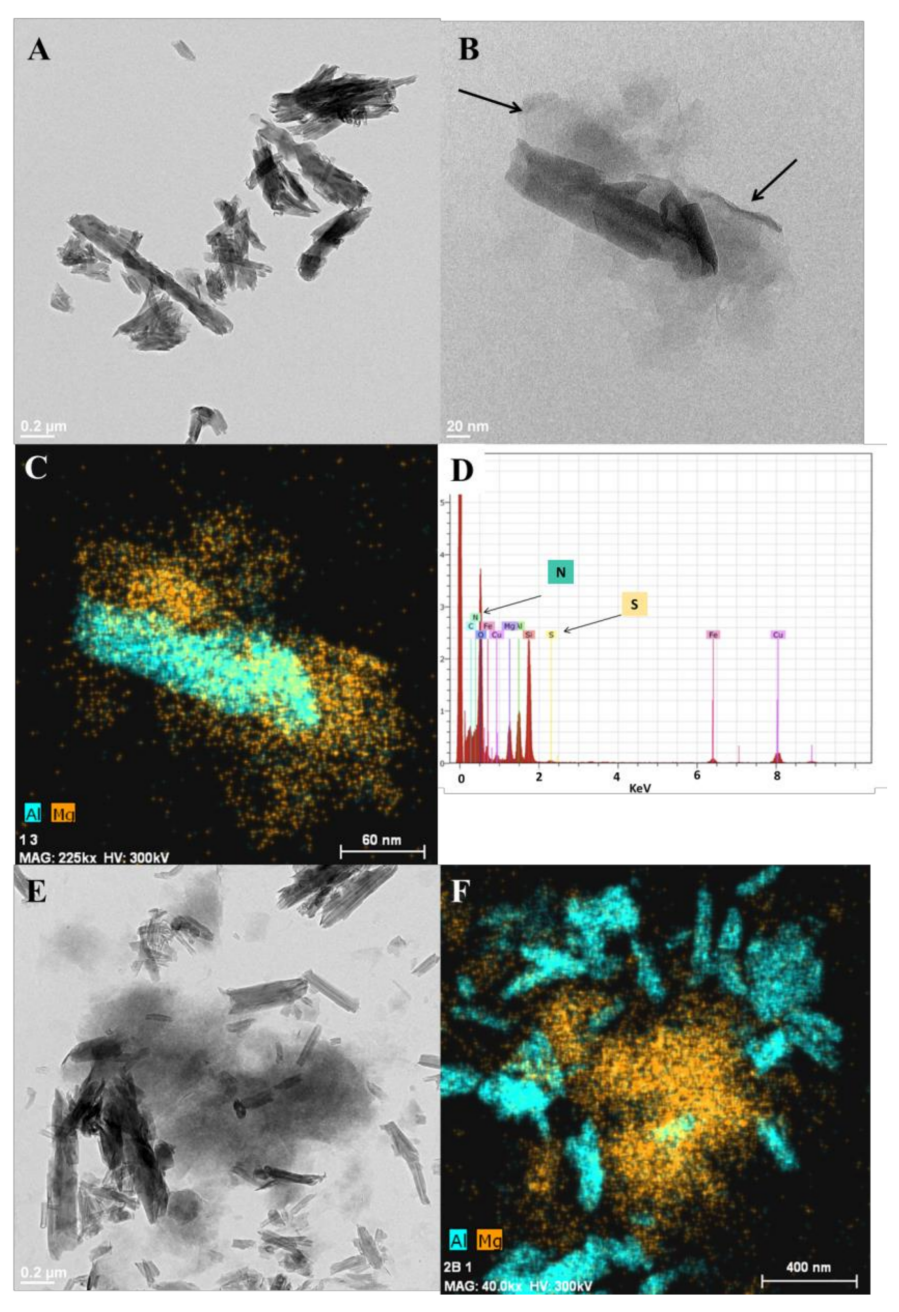
(**a**,**b**) TEM image of HNTs-Ht nanomaterial (scale bar 200 and 20 nm, respectively); (**c**) corresponding EDX image of Al and Mg from STEM image (scale bar 60 nm); (**d**) EDX spectrum of the HNTs-Ht nanomaterial; (**e**) TEM image of the physical mixture constituted by HNTs and Ht (scale bar 0.2 μm); and (**f**) the corresponding EDX image of Al and Mg from STEM image (scale bar 0.4 μm).

The hybrid material showed a rather compact structure with a dimension of ca. 135 nm (Figure 3a,b). Considering an halloysite diameter of ca. 100 nm and a Ht one of ca. 25 nm [6], the result is coherent with the two clays bonded. Close observations revealed the presence of halloysite nanotubes covered by hectorite clay (black arrow in the figure). Further confirmation was obtained by combining the high angle annular dark field scanning TEM (HAADF-STEM) and energy dispersive X-ray analysis. The former gives Z-contrast images allowing discrimination between the light and heavy atoms compounds, the latter allows chemical identifications of the atoms. By the combination of the two techniques a 2D-chemical map of the different elements may be extracted. In Figure 3c are reported the elemental X-ray aluminum and magnesium maps. In the hybrid HNTs-Ht material a magnesium layer (attributed to the hectorite) is covering the halloysite nanotubes (as highlighted by the presence of aluminum). 

EDX analysis (Figure 3 d and Figure S3) further confirmed the synthesis of the covalently linked clays highlighting the presence of all characteristic atoms of HNTs and Ht and the presence of sulfur and nitrogen in the nanomaterial deriving from the organic functionalities introduced. For comparison, we also acquired the HAADF-STEM (Figure 2e,f) of a physical mixture constituted by both clay precursors mixed in the same proportion used for the synthesis of HNTs-Ht nanomaterial. In this case the morphology of the mixture was different. Indeed, it is possible to distinguish the individual components, observing both halloysite nanotubes and hectorite randomly dispersed. 

**Figure 4:**
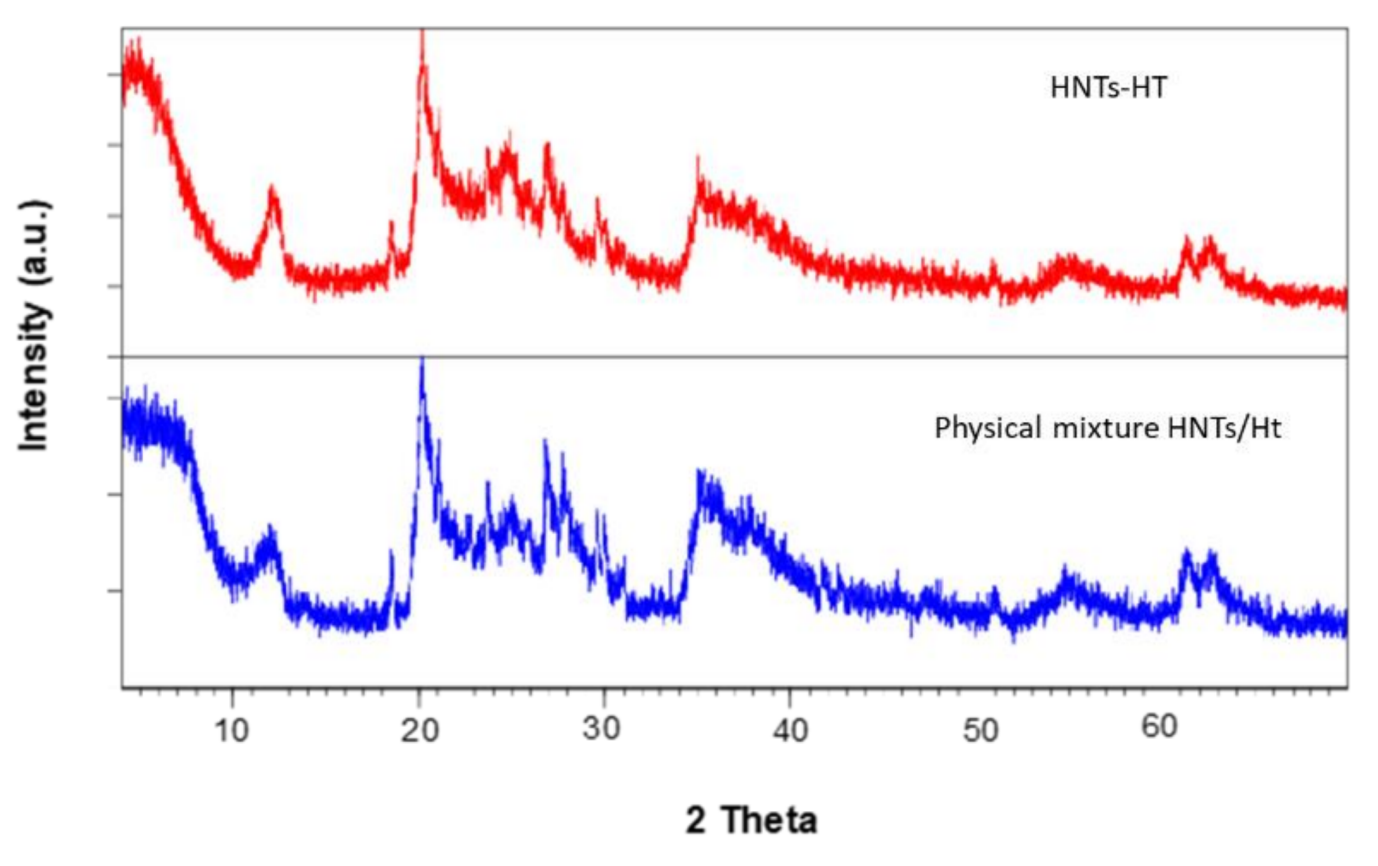
Comparison between the XRD patterns of HNTs/Ht physical mixture and HNTs-Ht nanomaterial.

To gather information about the structure of the HNTs-Ht nanomaterial, XRD measurements were performed on both pristine clays, HNTs-Ht and on its physical mixture (Figure 4). 

Both the physical mixture and HNTs-Ht nanomaterial show typical reflections of both HNTs and Ht [22]. After the covalent linkage between the two clays, all reflection peaks remain unchanged indicating that no structural variation occurs. Only an exception related to the basal reflection d_001_ of the hectorite was found. This shifts to lower 2θ values (ca. 4.84° 2θ) in comparison with the pristine clay mineral which showed a d_001_ basal reflection at 7.25° 2θ. These values indicated an increase in the interlayer space of Ht in the HNTs-Ht nanomaterial with respect to the pristine one, from 12.18 Å to 18.24 Å according to Bragg’s law, for pristine Ht and HNTs-Ht, respectively. This increase is due to the clay exfoliation as a consequence of the edges modification and a significant insertion of the silane coupling agent into the Ht interlayer according to previous reported for similar systems [23].

### 3.3. Adsorption Studies

As a proof of concept, the adsorption ability of HNTs-Ht nanomaterial in comparison to the pristine clays was investigated by adsorption experiments towards ciprofloxacin and silver ions chosen as models (details of the ciprofloxacin properties are reported in SI) (Table 3).

Experimental investigations performed by means of TGA and AgCl test (commonly adopted to estimate the amount of Ag^+^ ions in solution by exploiting the low solubility of AgCl salt) highlighted that ciprofloxacin did not interact with pristine Ht (entry 2), on the contrary, it was encapsulated into the pristine HNTs lumen (entry 1), whereas silver ions preferentially interact with Ht, presumable by cation exchange in the interlayer planes of the Ht particles as commonly reported for the clays belonging to the smectite group [24] (entry 2).

The HNTs covalently linked Ht showed the simultaneously adsorption of both ciprofloxacin and Ag^+^ ions (entry 3). The characterization of the drugs loaded nanomaterial highlighted the presence of ca. 2.6 wt% of ciprofloxacin and ca. 8 wt% of Ag^+^ ions, estimated by TGA (Figure S4) and AgCl test, respectively.

For comparison, the adsorption ability of the physical mixture of HNTs and Ht towards ciprofloxacin and Ag^+^ ions, was also investigated (entry 4). Conversely to HNTs-Ht nanomaterial, the physical mixture can efficiently adsorb Ag^+^ ions, whereas no loading of ciprofloxacin occurs. The latter could be explained by the different disposition of the clays in the two cases. In particular, in the physical mixture the clays, presumably, arrange themselves to minimize repulsive interactions; Ht nanodisks could locate adjacent to the ends of HNTs by blocking the lumen as reported in literature both in the case of HNTs dispersions at different pH and mixture HNTs/Laponite^®^ [25,26].

Therefore, it is possible to conclude that the HNTs-Ht nanomaterial, combining the properties of both clays, could be considered an efficient system for the co-delivery of species with different chemical characteristics.

## 4. Conclusions

In conclusion, we have reported a straightforward, simple and controllable method for the covalent linkage between two different clay minerals, namely halloysite nanotubes and hectorite. To reach this objective, hectorite was first functionalized in a two steps procedure. The physico-chemical characterization showed that pristine components retained their physico-chemical properties after the linkage. The morphology of the nanomaterial was imaged by TEM/HAADF technique. Finally, preliminary investigations were performed in order to verify the complementary properties of the nanomaterial in comparison to those of pristine clays towards the adsorption of ciprofloxacin and silver ions, chosen as models.

The designed nanomaterial demonstrated adsorption abilities similar to the pristine components separately, and consequently could be used as multi-functional or co-processed excipient, with improving manufacturing process of pharmaceutical products, including as for example, reduction of operating cost and improvement of final product quality. Furthermore, HNTs-Ht nanomaterial could be an alternative system for the simultaneous co-delivery of different antibacterial agents for the treatment of different patologies. It, indeed presents similar adsorption behavior of other systems commonly used, such as bentonite, fluorohectorite, montmorillonite, and hydroxyapatite, for the delivery of ciprofloxacin and silver ions [27,28,29,30,31].

## Figures and Tables

**Table 1:** Thermogravimetric parameters for HT, HT-NH_2_, and HT-Allyl.

Material	ML_150_ (wt%)	MR_800_ (wt%)	MD_800_ (wt%)
HT	11.09	82.0	6.91
HT-NH_2_	8.76	79.4	11.84
HT-Allyl	3.58	73.8	22.62

**Table 2:** Hydrodynamic diameter and the ζ−potential data.

Material	Hydrodynamic Diameter (nm)	ζ−Potential (mV)
Ht	432 ± 80	−36.9 ± 1.9
Ht-Allyl	484 ± 89	−15.0 ± 1.4
HNTs	421 ± 91	−34.4 ± 1.8
HNTs-SH	427 ± 95	−27.1 ± 1.1
HNTs-Ht	453 ± 96	−17.3 ± 1.4; −35.5 ± 1.7

**Table 3:** Adsorption ability of HNTs, Ht and HNTs-HT towards ciprofloxacin and Ag^+^ ions.

Entry	Sample	mg	Ciprofloxacin (wt%) ^a^	Ag^+^ ions (wt%) ^b^
1	HNTs	16	2.6	<1
2	Ht	16	<1	7
3	HNTs-Ht	32	2.6	8
4	HNTs/Ht	32	<1	7.6

^a^ Estimated by TGA, ^b^ Estimated by AgCl test.

## Data Availability

The data presented in this study are available on request from the corresponding author.

## Supplementary Materials

The following are available online at https://www.mdpi.com/2079-4991/11/2/506/s1, **Figure S1.** TGA curve of HNTs-Ht; **Figure S2.** TEM images of pristine HNTs and Ht; **Figure S3.** EDX spectrum of HNTs-Ht; physico-chemical properties of ciprofloxacin, **Figure S4.** TGA curve of HNTs, Ht and HNTs-Ht loaded with ciprofloxacin.
