# Supplementary material for: Synthesis and Characterization of Nanomaterial Based on Halloysite and Hectorite Clay Minerals Covalently Bridged
Source: Nanomaterials (Basel). 2021 Feb 17;11(2):506. doi: 10.3390/nano11020506 (PMC7922904; doi:10.3390/nano11020506)
Supplement: Supplementary file 1 [file nanomaterials-11-00506-s001.pdf]

nanomaterials

Supply

Synthesis and Characterization of Nanomaterial Based on Halloysite and Hectorite Clay Minerals Covalently Bridged

Marina Massaro [1], Cesar Viseras Iborra [2,3,*], Giuseppe Cavallaro [4,5], Carmelo Giuseppe Colletti [1], Fátima García-Villén [2], Giuseppe Lazzara [4,5] and Serena Riela [1,*]

[1] Department of Biological, Chemical and Pharmaceutical Sciences and Technologies, University of Palermo Viale delle Scienze, Ed. 17 90128 Palermo, Italy; marina.massaro@unipa-it (M.M.); carmelogiuseppe.colletti@unipa.it (C.G.C.)
[2] Department of Pharmacy and Pharmaceutical Technology, Faculty of Pharmacy. University of Granada, Campus of Cartuja, 18071 s/n Granada, Spain; fgarvillen@ugr.es
[3] Andalusian Institute of Earth Sciences, CSIC-UGR. Avenida de las Palmeras 4, 18100 Armilla, Granada, Spain
[4] Department of Physics and Chemistry, University of Palermo, Palermo 90128, Italy; giuseppe.cavallaro@unipa.it (G.C.); giuseppe.lazzara@unipa.it (G.L.)
[5] National Interuniversity Consortium of Materials Science and Technology (INSTM), Research Unit of Palermo, Via G. Giusti, 9, 50121 Florence, Italy
* Correspondence: cviseras@ugr.es (C.V.I.); serena.riela@unipa.it (S.R.)

Citation: Massaro, M.; Iborra, C.V.; Cavallaro, G.; Colletti, C.G.; García-Villén, F.; Lazzara, G.; Riela, S. Synthesis and Characterization of Nanomaterial Based on Halloysite and Hectorite Clay Minerals Covalently Bridged. *Nanomaterials* **2021**, *11*, 506. https://doi.org/10.3390/nano11020506

Academic Editor:
Received: 26 January 2021
Accepted: 15 February 2021
Published: date

Publisher's Note: MDPI stays neutral with regard to jurisdictional claims in published maps and institutional affiliations.

Copyright: © 2021 by the authors. Submitted for possible open access publication under the terms and conditions of the Creative Commons Attribution (CC BY) license (http://creativecommons.org/licenses/by/4.0/).

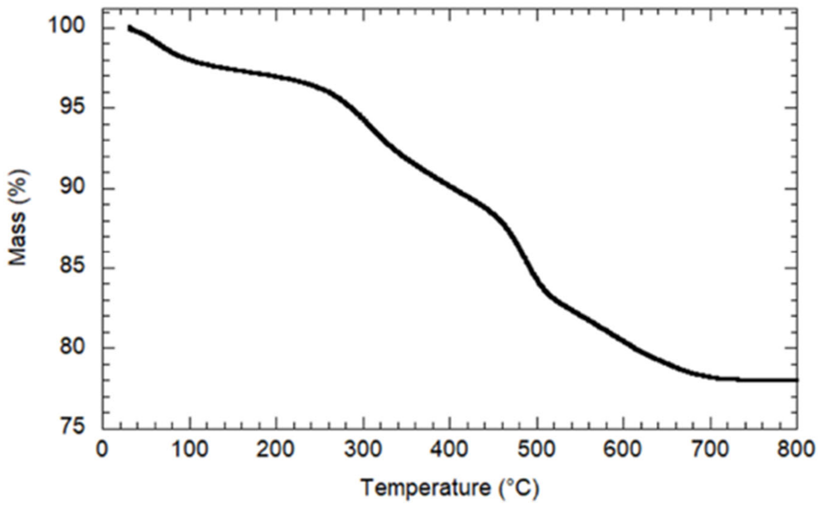

Figure S1. Thermogravimetric curve for HNTs-Ht nanomaterial.

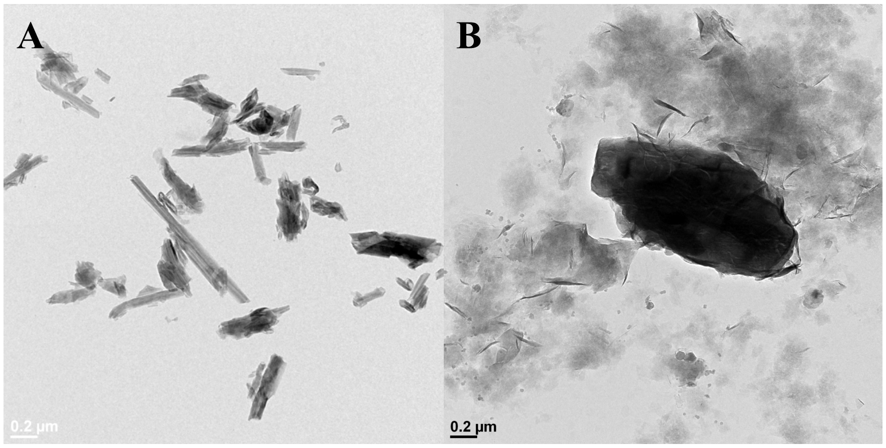

Figure S2. TEM images of (a) pristine HNTs; (b) pristine Ht.

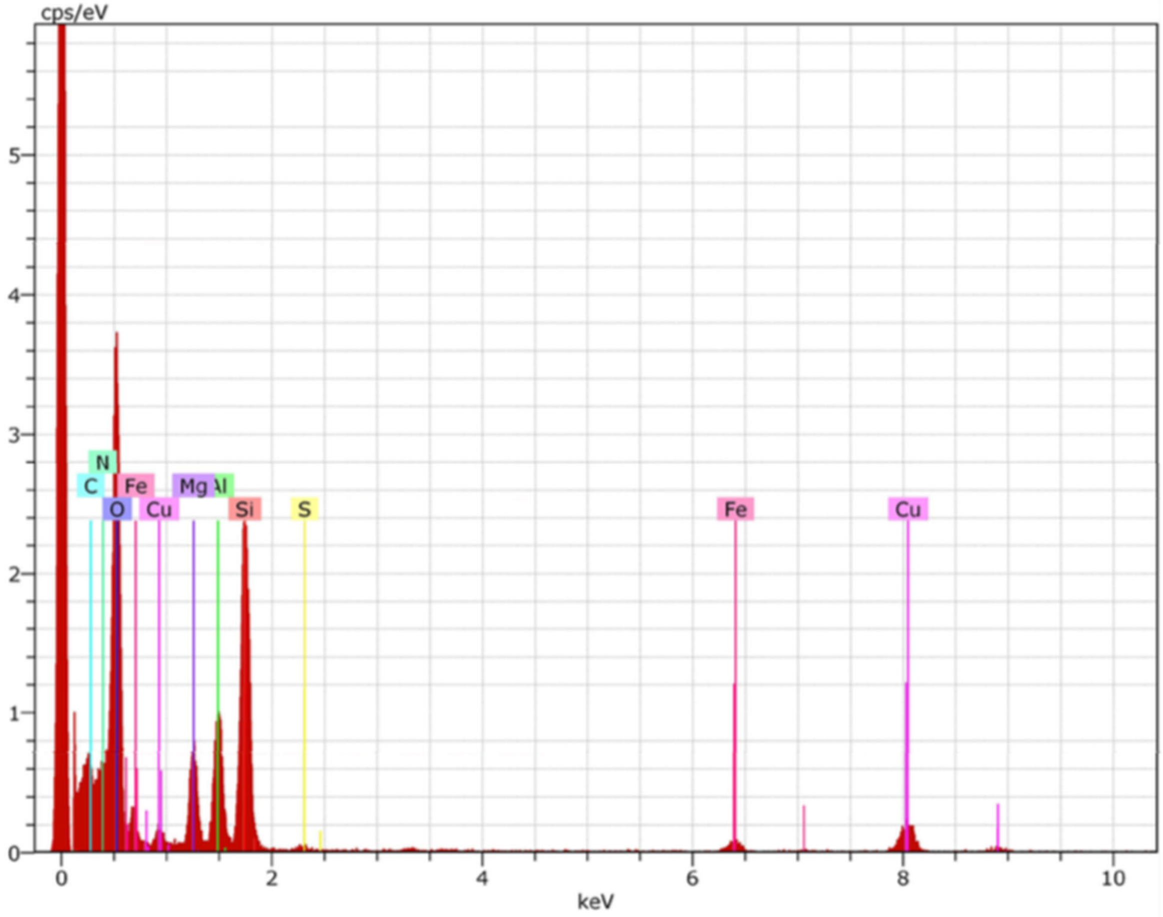

Figure S3. EDX spectrum of the HNTs-Ht nanomaterial.

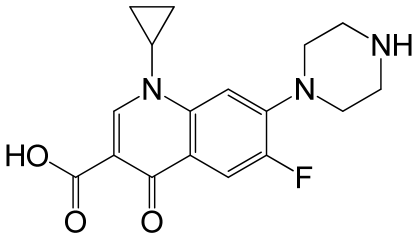

Ciprofloxacin is widely used as antibiotics to treat several types of inflammations including skin infections since they are among the most used broad-spectrum antibiotics active against both Gram-positive and Gram-negative bacteria (Campoli-Richards et al., 1988).

Ciprofloxacin is a zwitterionic molecule which possesses a pH-dependent speciation (pK_{a1} = 6.1 and pK_{a2} = 8.7), it is soluble in acidic aqueous medium, but it is insoluble in alcohols. Therefore, ciprofloxacin exists mainly in cationic form at pH < 4.0, in cationic and zwitterionic form at 4.0 < pH < 8.0 and in anionic form at pH > 8.0 (Li et al., 2017).

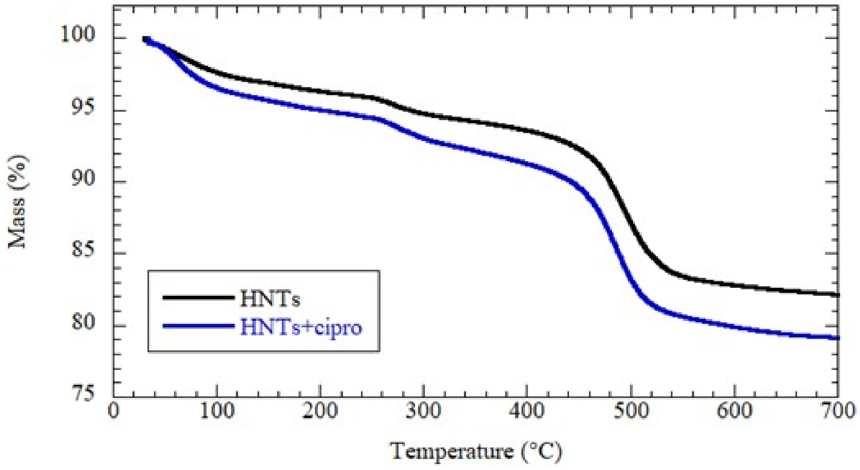

(a)

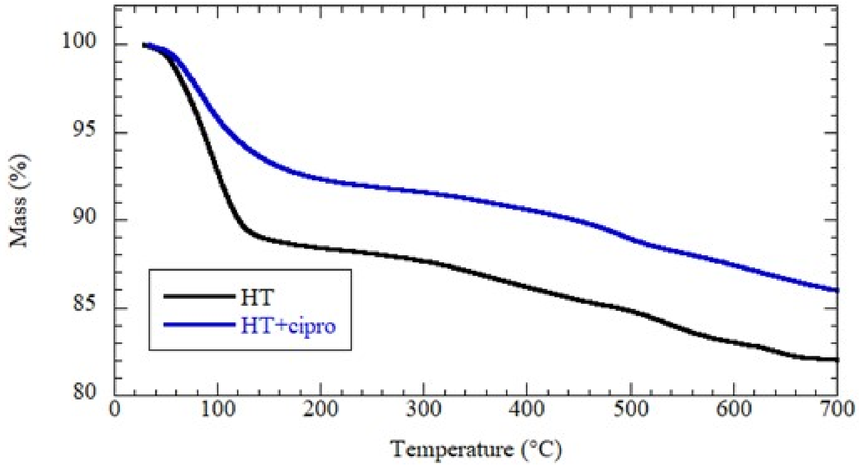

(b)

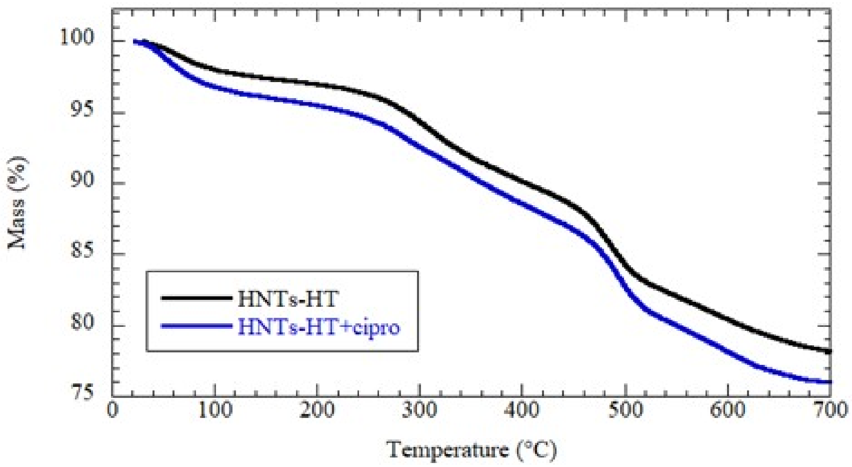

(c)

Figure S4. Thermogravimetric curves of (a) HNTs, (b) Ht and (c) HNTs-HT nanomaterials loaded with ciprofloxacin.

References

1. Campoli-Richards, D.M., Monk, J.P., Price, A., Benfield, P., Todd, P.A., Ward, A., 1988. Ciprofloxacin. Drugs 35, 373-447.
2. Li, S., Zhang, X., Huang, Y., 2017. Zeolitic imidazolate framework-8 derived nanoporous carbon as an effective and recyclable adsorbent for removal of ciprofloxacin antibiotics from water. Journal of Hazardous Materials 321, 711-719.
